# Errata corrige

**Published:** 2019-01-20

The manuscript “siRNA Delivery for Control of Cyclin D1 and E2F1 Expression in Crohn’s Disease” published in Transl Med UniSa. 2018 Mar 31;17:25–33, reports a mistake in the affiliation list. The correct affiliation for dott Albino Carrizzo is: IRCCS Neuromed, Pozzilli (IS), 86077, Italy and for prof Vecchione is Department of Medicine and Surgery, Scuola Medica Salernitana, University of Salerno, 84081, Baronissi (SA), Italy, and IRCCS Neuromed, Pozzilli (IS), 86077, Italy.

